# Shrinking Lung Syndrome: A Case Report

**DOI:** 10.7759/cureus.27311

**Published:** 2022-07-26

**Authors:** Asma Haji Datoo, Loui Abdelghani

**Affiliations:** 1 Internal Medicine, St. Joseph’s Medical Center, Stockton, USA; 2 Pulmonary and Critical Care Medicine, St. Joseph’s Medical Center, Stockton, USA

**Keywords:** diaphragm injury, respiratory paralysis, diaphragm dysfunction, systemic lupus erythematous disease, shrinking lung syndrome

## Abstract

Shrinking Lung Syndrome (SLS) is an uncommon complication of systemic lupus erythematosus (SLE). SLS is a diagnosis of exclusion with features of dyspnea ruled out by other causes using imaging and diagnostic studies, pleuritic chest pain, and elevated diaphragm. Currently, there are many theories of the etiology; however, there is no clear pathogenesis, conclusive treatment, and preventative measures. We report a case of a 41-year-old woman with SLE admitted for pleuritic chest pain with unclear cause of shortness of breath. After CTA chest study, laboratory, chest x-ray, and pulmonary function test we were able to appropriately diagnose her with SLS and treat her with steroids as per limited current research guidelines.

## Introduction

Shrinking lung syndrome (SLS) is a rare complication of systemic lupus erythematous (SLE) that effects less than 1% of patients. SLS has been seen with other autoimmune disorders [1]. Unfortunately, the underlying pathophysiology is unclear. Many theories include pleural inflammation, phrenic neuropathy, pleural adhesion, and myopathy [2]. Symptoms include shortness of breath, chest pain, normal lung parenchyma, restrictive ventilatory defect, and elevation of the diaphragm. We describe a patient with SLE admitted for shortness of breath with pulmonary function test (PFT) and other studies diagnosing her with SLS.

## Case presentation

A 41-year-old woman with a past medical history of pericarditis with pericardial window, pulmonary hypertension with World Health Organization (WHO) Group I secondary to SLE functional classification II on two liters home oxygen, SLE, congestive heart failure (CHF), chronic kidney disease (CKD), and hypertension presented to the hospital complaining of chest pain and worsening shortness of breath requiring four liters of oxygen. The patient complained of chronic non-radiating chest pain due to pericarditis, which was worsening for one day in the left upper chest. The patient denied any palpitations, dizziness, cough, fevers, chills, nausea, vomiting, or diarrhea. At baseline, the patient could complete her daily chores with two liters of oxygen but was having difficulty breathing at the time while taking a few steps to get to the bedside commode. The patient was following up at the pulmonary hypertension clinic. The last visit was several months ago, and she reported being stable on ambrisentan 10mg daily and tadalafil 40mg daily. 

Her initial vital signs in the emergency department were 36.8 degrees Celsius, 103 beats per minute, respiratory rate of 22, blood pressure 139/102 mmHg, 2-4 liters of oxygen saturating at 95-99%. Complete blood count was unremarkable, erythrocyte sedimentation rate 34 mm/hr, complete metabolic panel unremarkable except for alkaline phosphatase of 163 units/L, c-reactive protein 1.03mg/dL, COVID-19 negative. Prior serological labs from admission were remarkable for antinuclear antibody (ANA), chromatin QN, double-stranded DNA (dsDNA), anti-Smith (sm), Smith QN, sm/ribonucleoprotein (RNP), Anti-U1RNP.

**Figure 1:**
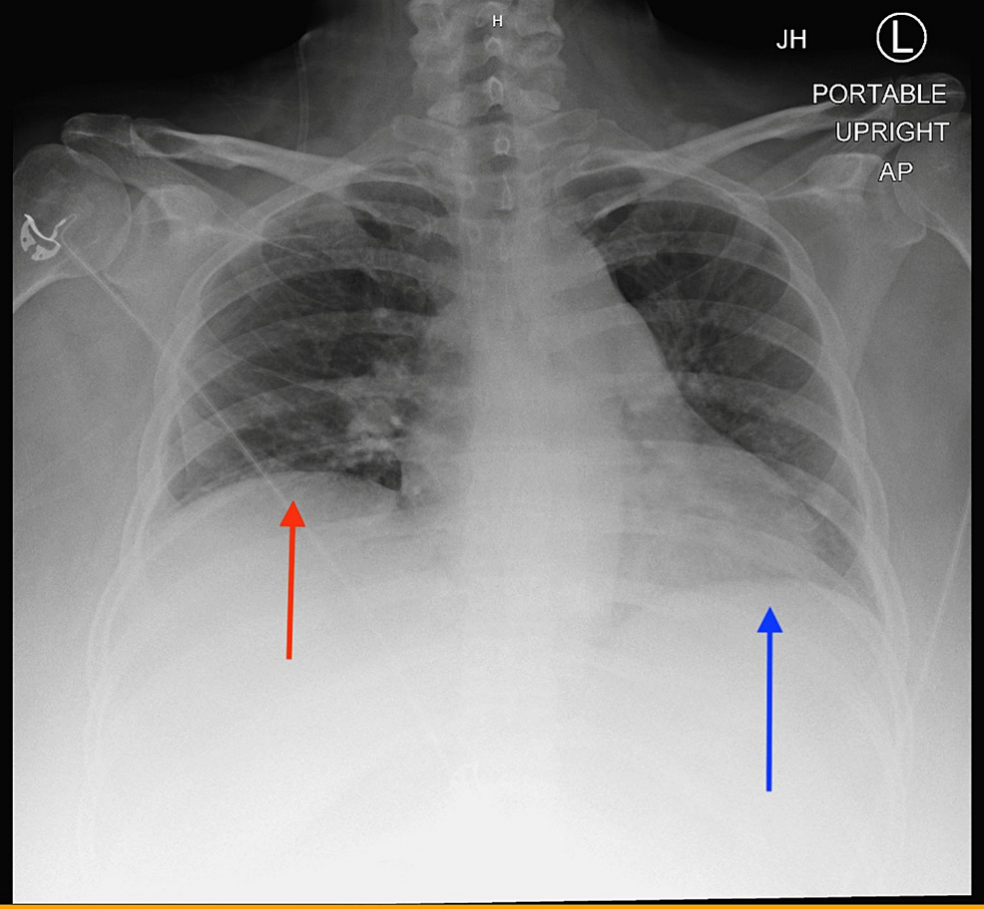
Right side (red arrow) demonstrating elevated diaphragm compared to the left side (blue arrow)

**Figure 2:**
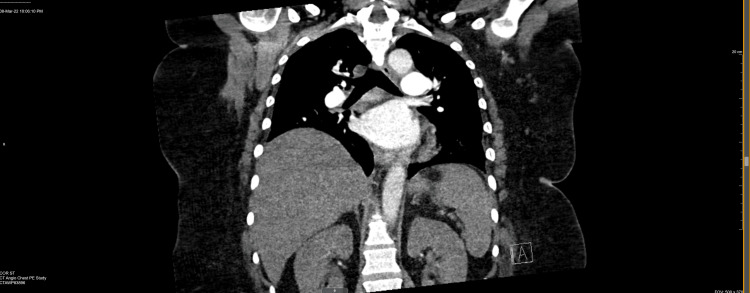
Computed tomography scan chest angiogram (coronal section) demonstrating right side diaphragm elevation

**Figure 3:**
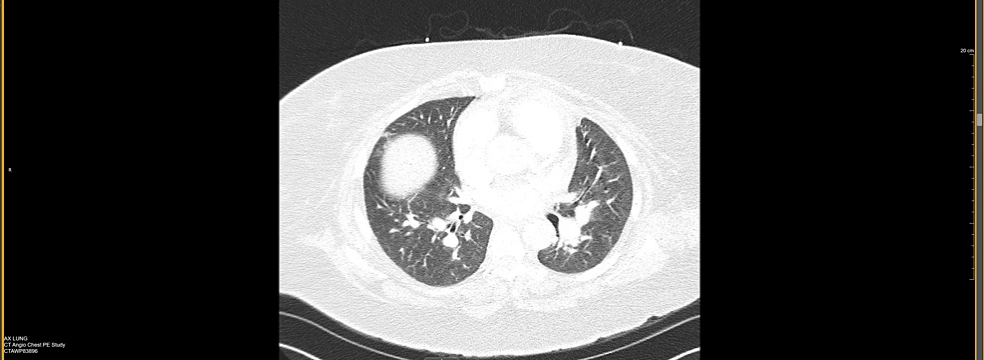
Computed tomography scan of chest angiogram in transverse section reveals right-sided hemidiaphragmatic elevation

A chest x-ray taken during the inspiratory phase revealed bibasilar opacities that could reflect atelectasis and a right elevated diaphragm (Figure 1). CT angiogram of the chest showed prominent pulmonary main and lobar arteries. No intraluminal filling defect was observed in the main, lobar, segmental, or subsegmental pulmonary arteries. The diameter of the thoracic aorta and course was normal. Both lungs were clear without pleural effusion, pneumothorax, patent trachea, and central bronchi. The heart was not enlarged. No pericardial effusion or lymphadenopathy was present (Figures 2, 3).

Transthoracic echocardiogram showed an ejection fraction of 55%, right ventricle cavity size was severely dilated, and pressure during systole by Doppler was 52 mmHg. Prior right ventricle pressure was 46mmHg. The ventricular septum had diastolic flattening, which is consistent with right ventricle volume/pressure overload. The pulmonary function test demonstrated severe restrictive lung disease, reduced total lung capacity (TLC), and reduced diffuse capacity of the lung for carbon monoxide (DLCO) (Table 1).

**Table 1:** Pulmonary function test demonstrating a non-obstructive pattern with low DLCO FEV1: forced expiratory volume in the first second of forceful expiration; FVC: forced vital capacity; TLC: total lung capacity; DLCO: diffuse capacity of lung for carbon monoxide; FEF: forced expiratory flow; FIVC: forced inspiratory vital capacity; FIF: forced inspiratory flow; SVC: slow vital capacity; IC: inspiratory capacity; ERV: expiratory reserve volume; TGV: thoracic gas volume; RV (Pleth): residual volume; DLCOunc: diffusing capacity of the lungs for carbon monoxide uncorrected; DLCOcor: diffusing capacity of the lungs for carbon monoxide corrected; DLCO: diffusing capacity of the lungs for carbon monoxide; VA: alveolar volume; GAW: airway conductance; sRaw: specific airway resistance; sGaw: specific airway conductance FEV1/FVC is greater than 70%, which means this is a non-obstructive finding in spirometry. Low FEV1, FVC, TLC, and DLCO: this pattern demonstrates severe restrictive disease.

Pre-bronchodilator			
	Actual	Pred	% Pred
Spirometry			
FVC (L)	1.17	3.51	33
FEV1 (L)	1	2.73	36
FEV1/FVC(%)	86	78	36
FEF 25% (L/sec)	3.03		
FEF 75% (L/sec)	0.43	1.08	39
FEF 25-75% (L/sec)	1.23	3.15	39
FEF Max (L/sec)	3.69	5.38	68
FIVC (L)	1.12		
FIF Max (L/sec)	2.43		
Lung volumes			
SVC (L)	1.19	3.44	34
IC (L)	0.9	2.25	40
ERV (L)	0.29	1.19	24
TGV (L)	1.89	2.8	67
RV (Pleth) (L)	1.61	1.61	99
TLC (Pleth) (L)	2.8	5.05	55
RV/TLC (Pleth) (%)	57	32	179
Trapped gas (L)			
Diffusion			
DLCOunc (ml/min/mmHg)	5.46	27.49	19
DLCOcor (ml/min/mmHg)		27.49	
DL/VA (ml/min/mmHg)	3.26	5.44	59
VA (L)	1.67	5.05	33
Airway Resistance			
Raw (cmH20/L/s)	1.03	1.86	55
GAW (L/s/cmH20)	0.97	1.03	94
sRaw (cmH20*s)	1.92	<4.76	
sGaw (1/cmH20*)	0.52	0.2	260

The patient appeared comfortable, oriented to name, birthday, location, situation, S1S2 heart sound, lungs clear to auscultate bilaterally, abdominal examination unremarkable, no pitting edema to extremities, and well perfused. The patient’s ambrisentan and tadalafil were resumed during the hospitalization and prednisone was initiated. The patient was in the hospital for a total of five days and her oxygenation improved and was appropriately discharged. 

## Discussion

Most patients with SLE eventually show symptoms of respiratory involvement. Coughing and dyspnea are the first clues of SLE pulmonary development [3]. The most common presentation is pleuritis. Other manifestation includes interstitial lung disease (ILD), acute lupus pneumonitis, diffuse alveolar hemorrhage, pulmonary arterial hypertension, acute reversible hypoxemia, and SLS [4]. SLS is diagnosis of exclusion and is characterized by respiratory muscle dysfunction such as the diaphragm causing shortness of breath, pulmonary function test with restrictive lung patterns, elevated diaphragm on chest x-ray, and no parenchymal or vascular obstruction such as pulmonary embolism on CT study [5,6]. A possible differential diagnosis is pulmonary hypertension, however the pulmonary function test would show decreased DLCO and not a reduction in TLC. To appropriately diagnose SLS, one must first rule out the common conditions causing dyspnea such as pneumonia, viral or bacterial pleurisy, pericarditis, and pulmonary embolism [2]. Once SLS is diagnosed, treatment is steroid therapy. Typical dose is 0.5mg-1.0mg/kg of prednisolone. Unfortunately, complete recovery of clinical, functional, and radiographic abnormalities is uncommon; however, with steroid therapy, SLS has good prognosis, improvement in pulmonary function tests, and symptomatic relief [2]. There are several reports that also mention that the use of azathioprine, rituximab, cyclophosphamide, methotrexate, theophylline, and beta-agonist may aid in symptomatic relief [1].

## Conclusions

Our patient with SLE, admitted for worsening shortness of breath, pleuritic chest pain, was diagnosed with SLS. We appropriately treated her with steroids after ruling out other causes of the dyspnea. Pathogenesis for SLS is still undefined and further research is warranted. Clinicians should be mindful of SLS when dealing with a patient with shortness of breath and an autoimmune disease.

SLS is rare, possibly due to being underreported. The hint to SLS is when the underlying cause of shortness of breath is not clear with a raised hemidiaphragm. Unilateral or bilateral elevation of the hemidiaphragm and basal atelectasis is ubiquitous in this condition. Currently, the data we have supports the use of steroids, which does have positive outcomes.
